# Diet Quality and Cognitive Performance in Children Born Very Low Birth Weight

**DOI:** 10.3389/fnut.2022.874118

**Published:** 2022-07-19

**Authors:** Julie Sato, Meghan McGee, Nicole Bando, Nicole Law, Sharon Unger, Deborah L. O'Connor

**Affiliations:** ^1^Department of Diagnostic Imaging, The Hospital for Sick Children, Toronto, ON, Canada; ^2^Department of Psychology, University of Toronto, Toronto, ON, Canada; ^3^Neuroscience and Mental Health Program, The Hospital for Sick Children Research Institute, Toronto, ON, Canada; ^4^Department of Nutritional Sciences, University of Toronto, Toronto, ON, Canada; ^5^Department of Translational Medicine, The Hospital for Sick Children Research Institute, Toronto, ON, Canada; ^6^Department of Paediatrics, University of Toronto, Toronto, ON, Canada; ^7^Department of Paediatrics, Sinai Health, Toronto, ON, Canada; ^8^Division of Neonatology, The Hospital for Sick Children, Toronto, ON, Canada

**Keywords:** preterm, very low birth weight, diet quality, cognition, children

## Abstract

Children born very low birth weight (VLBW, <1,500 g) are at high risk for cognitive and academic difficulties later in life. Although early nutrition (e.g., breastfeeding) is positively correlated with IQ in children born VLBW, the association between dietary intake in childhood and cognitive performance is unknown. Thus, our study is the first to investigate the relationship between diet quality, as measured by the Healthy Eating Index-2010 (HEI-2010) and cognitive performance in a Canadian cohort of 5-year-old children born VLBW (*n* = 158; 47% female). Diet quality was measured using two 24-h diet recalls obtained from parents and cognitive performance was assessed using the Wechsler Preschool and Primary Scale of Intelligence-IV (WPPSI-IV). To account for additional sociodemographic factors that could influence neurodevelopment, linear regression analyses were adjusted for sex, household income above/below the poverty line, maternal education, birth weight and breastfeeding duration. Mean ± SD HEI-2010 score was 58.2 ± 12.4, with most children (67%) having diets in “need of improvement” (scores 51–80). HEI-2010 scores were not significantly associated with IQ or any other WPPSI-IV composite score. Significant predictors of IQ in our model were birth weight, sex, and maternal education. Our findings emphasize the important role of maternal education and other sociodemographic factors on neurodevelopment in children born VLBW. Further, despite not finding any significant association between HEI-2010 scores and IQ, our results highlight the need to improve diet quality in young children born VLBW. Further research is needed to confirm the impact of diet quality on cognitive performance in this vulnerable population.

## Introduction

Very low birth weight (VLBW, <1,500 g) infants are born early in the third trimester of pregnancy, a critical period of brain development, putting them at higher risk of cognitive impairments and academic underachievement later in life (1–3). Convergent findings support overall lower academic performance and IQ scores in preterm [<37 weeks gestational age (GA)] compared to full-term children, with one meta-analysis reporting almost a 12-point difference in IQ among school-aged children born preterm, independent of socioeconomic status [SES; (4)]. Several factors, as a consequence of preterm birth, increase the risk of sub-optimal neurodevelopment, including inadequate nutrient intake and morbidity during initial hospitalization [e.g., sepsis, necrotizing enterocolitis, chronic lung disease; (5–7)]. After hospital discharge, SES factors play a central role in the neurodevelopment of preterm infants, but other factors such as childhood nutrition have not yet been explored (8, 9).

While the role of post-discharge diet on neurodevelopment in infants born VLBW is unknown, evidence from studies of full-term infants suggests that a moderate association exists between diet and academic outcomes (8). In full-term children, different components of the diet (e.g., fruits and vegetables, breakfast consumption) are associated with cognition (8, 10, 11). In one large Canadian cohort (*n* = 5,200) of grade 5 students, higher diet quality scores, assessed by the Diet Quality Index-International and Healthy Eating Index (HEI), were associated with a reduced likelihood of failing school literacy assessments (12). More recently, a cross-sectional analysis in a Spanish cohort of children and adolescents (*n* = 1,371, 8–18 years) found better adherence to a Mediterranean diet, as measured by the KIDMED diet quality index, was associated with higher academic performance (13). A Mediterranean diet is typically rich in vegetables, fruit, nuts, fish and olive oil. Importantly, these findings remained significant after adjusting for various confounders including maternal education, sex, age, birth weight and GA. Infants born VLBW, however, were not included in these studies.

In preterm and VLBW children, the only studies investigating the relation between diet and cognition have focused on nutrient intake during early infancy. In one study, greater breastmilk intake during initial hospitalization in preterm infants (<37 weeks GA) was associated with higher verbal IQ in adolescence [*n* = 50, 13–19 years; (14)]. In boys, breastmilk intake was also linked with higher IQ scores, as well as total brain and white matter volume. Importantly, in the seminal meta-analytic paper by Anderson et al. (1), the authors found that low birth weight infants who were breastfed derived a greater cognitive benefit than normal birth weight infants, even after adjusting for covariates such as SES or maternal education. More recent systematic reviews and a cluster-randomized control trial also support the positive association between breastfeeding and neurodevelopment that persists after adjustment for maternal IQ in both full-term and preterm children (15, 16).

Given the high risk of academic underachievement and cognitive difficulties in children born VLBW, elucidating modifiable factors that impact cognitive outcomes in this population is an essential step for informing supports and strategies for families and educators (17, 18). Further, preschool age represents a critical time period to investigate the association between diet and cognitive development, as dietary preferences are established early in childhood (19), and cognitive ability at this age is associated with later academic achievement in full-term children (20, 21). Thus, our study estimated diet quality in children born VLBW using the HEI-2010 (22, 23) to evaluate associations with cognitive performance. In line with the full-term literature, we hypothesized that higher HEI-2010 scores (reflecting closer conformance with dietary guidelines) would correspond with higher IQ scores.

## Methods

### Participants

All surviving children and their families who consented to the original randomized trial were approached for the present follow-up study when the children were 5 years of age. The feeding intervention, study protocol and outcomes have been published (24–27). Briefly, VLBW infants were enrolled between October 2010 and December 2012, from four tertiary neonatal intensive care units in Ontario, Canada. Infants were eligible if their birth weight was <1,500 g, and if families consented within 4 days of birth. Exclusion criteria included chromosomal or congenital anomalies that could affect neurodevelopment. At the 5-year follow-up, written informed consent was obtained from parents and verbal assent from children.

### Exposures: Diet Quality, Sociodemographic and Lifestyle Characteristics

A 24-h dietary recall was completed with parents during the study visit and parents were contacted 1 week later to complete a second recall by phone or email as described in detail previously (28). Briefly, the 24-h dietary recall asks parents to detail everything their child ate and drank in the previous day using the Automated Self-Administered 24-h (ASA24) Dietary Assessment tool, Canadian version (2016). The HEI-2010, a validated measure of diet quality in both children and adults (22, 23), was used to assess diet quality. Importantly, this tool considers diet quality distinctly from the quantity of food consumed (i.e., food groups are considered per 1000 kcal consumed).

The HEI-2010, created by the U.S. Department of Agriculture, consists of 12 components: nine adequacy components (that should be consumed in adequate amounts for optimal health) and three moderation components (that should be consumed in limited amounts for optimal health; see Supplementary Tables 1, 2 for the HEI-2010 Components and Standards for Scoring). Overall diet quality is reflected in the total index score, which is the sum of the 12 dietary components. HEI-2010 scores range from 0 to 100, with higher scores reflecting greater adherence to dietary requirements and better diet quality. Diets with HEI-2010 scores >80 are categorized as being “healthy”, with scores between 51 and 80 reflecting diets that “need improvement”, and scores <51 considered “poor” diets (22). HEI-2010 scores were calculated from the two non-consecutive 24-h dietary recalls using the multivariate Markov Chain Monte Carlo approach, which adjusts for weekday vs. weekend, season and recall sequence. A validated scoring algorithm was used to calculate individual HEI-2010 scores.

### Outcome Measures: Cognitive Performance

The primary outcome measure for this study was cognitive performance assessed using Full-Scale IQ on the Wechsler Preschool and Primary Scale of Intelligence-IV [WPPSI-IV; (29)]; the version based on Canadian norms. The WPPSI-IV is a standardized measure of intelligence for children between 2 years and 6 months through to 7 years and 7 months of age. Secondary outcome measures included the following composite scores of the WPPSI-IV: verbal comprehension index (VCI), vocabulary acquisition index (VAI), visual spatial index (VSI), fluid reasoning index (FRI), processing speed index (PSI), and working memory index (WMI). All WPPSI-IV composite scores are calculated as standard scores with a mean of 100 and a standard deviation of 15, with “low average” scores defined as scores <90 (29). In line with other experts in the field, children who were unable to complete the WPPSI-IV assessment due to severe disability or who performed below the threshold of the test, were assigned a score of 49 (24, 30–32).

### Demographic and Clinical Variables

Demographic and clinical factors including sex, birth weight, household income above/below the poverty line, maternal education, maternal ethnicity, and duration of breastfeeding were collected as described in detail previously (24). Briefly, sex and birth weight were collected from medical records and maternal education and ethnicity, as well as household income were reported from standardized parent questionnaires at birth. Maternal education was dichotomized as mothers having a university degree or not and household income above/below the poverty line was based on 2006 Statistics Canada family size-adjusted cut-off values (33). Duration of breastfeeding was calculated as the last recorded date that any breastmilk was provided, up to 18 months corrected age. During initial hospitalization, daily volumes of both parenteral and enteral nutrition were prospectively recorded in all infants. Enteral feed type during the intervention was characterized as receiving >50% of enteral feeds as either donor milk, preterm formula or mother's breastmilk.

### Statistical Analyses

Normality of data were confirmed visually using histogram distributions and analytically using the Shapiro-Wilk test. Normally distributed variables were described as mean (standard deviation or 95% confidence interval [CI]) or median (interquartile range) when not normally distributed. Independent samples *t*-tests were used to compare differences between sexes for continuous outcome variables, and categorical variables were compared using chi-square tests.

Linear regression models were computed for primary and secondary outcomes. The association between HEI-2010 scores and WPPSI-IV composite scores was analyzed using linear regression models adjusted for sex, birth weight, breastfeeding duration, income above/below the poverty line, and maternal education (university degree or not). Interactions between exposure variables and sex were assessed in linear regression models to test whether the association between diet quality and IQ varied by sex. If the interaction was not statistically significant, it was removed from the model and the analyses were re-run. Multicollinearity was assessed using variance inflation factors with a cut-off value >10 considered to represent multicollinearity. Statistical analyses were performed in R 3.5.1 (34) using the rms statistical package. All hypothesis tests were two-sided and considered statistically significant if *p*-values were <0.05.

### Sensitivity Analyses

To ensure that imputation of scores did not impact study findings, sensitivity analyses were performed by excluding children who were assigned a score of 49. In addition, in-hospital enteral feed type (receiving >50% mother's breastmilk, donor milk, or preterm formula) was included as a covariate in multivariable models to test for the effect of supplemental milk provided in-hospital. We also tested the interaction between enteral feed type and maternal education (university degree or not) in linear regression models. If the interaction was not statistically significant, it was removed from the model.

## Results

### Participant Characteristics

One hundred and fifty-eight of 316 (50% follow-up rate) eligible children born VLBW participated in the 5-year follow-up [mean (SD) age: 5.7 (0.2) years]. Birth and parental baseline characteristics are summarized in Table 1, including sex, birth weight, birth GA, mother's ethnicity, mother's education, family income, enteral feed type, and breastfeeding duration. Mean (SD) birth weight and GA were 1013 (264) g and 28 (3) weeks, respectively. Fifty percent of mothers in our sample had an education level of university or above and 20% had incomes below the poverty line. As reported previously, except for maternal education, no statistically significant differences in baseline characteristics were found between children who participated in the 5-year follow-up and those who did not (28). Mothers of children who participated in the follow-up study were more educated than mothers of children who did not (*p* = 0.02).

**Table 1:** Birth and parental baseline characteristics of children and families who participated in follow-up at 5 years.

**Characteristic**	***n***	**Mean (SD) or *n* (%)**
**Sex**		
Female	158	74 (47)
Male		84 (53)
Birth weight, grams	158	1,013 (264)
Birth gestational age, weeks	158	28 (3)
**Mother's ethnicity**		
European	156	68 (44)
Asian		25 (16)
Middle Eastern or South Asian		29 (19)
Mixed or other		34 (22)
**Mother's education**		
None/high school diploma	156	31 (20)
College/vocational diploma		41 (26)
Baccalaureate		49 (31)
Post baccalaureate		35 (22)
**Family living below poverty line**		
No	149	120 (81)
Yes		29 (19)
**Enteral feed type during initial hospitalization**		
>50% donor milk	158	22 (14)
>50% preterm formula		20 (13)
>50% mother's breastmilk		116 (73)
Breastfeeding duration, days	158	223 (181)

### Cognitive Assessments

**Figure 1:**
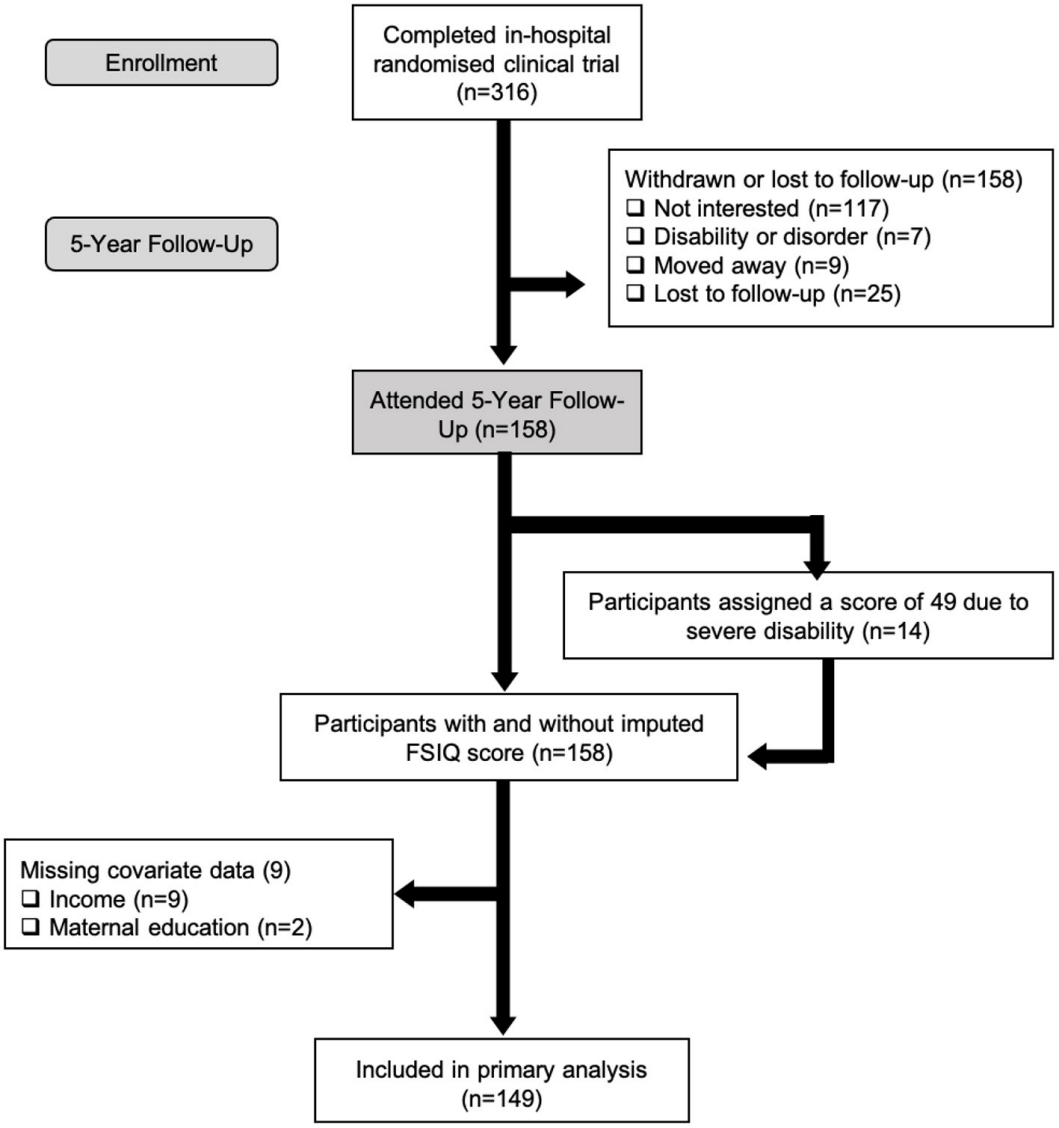
Flow of VLBW participants through the randomized clinical trial from initial enrollment to inclusion in primary analysis.

Complete WPPSI-IV data were obtained for 91.1% (144 of 158) of children born VLBW. Fourteen children who attended the follow-up visits were unable to complete the WPPSI-IV (see Figure 1 for participant flowchart). In these cases, an IQ score of 49 (the lower limit of IQ) was assigned. The mean (SD) of IQ was 94.3 (20.4), which is within the average range of standard IQ classification (90–109). The mean (SD) for all WPPSI-IV composite scores are summarized in Table 2. Males had significantly lower scores for IQ, FRI, WMI, and PSI. In addition, more males had an IQ score below 90, which according to the WPPSI-IV manual (29) is indicative of low average or borderline level cognitive ability (*p* = 0.006).

**Table 2:** WPPSI-IV composite scores for 5-year-old children born VLBW.

**WPPSI-IV composite scores**	**All participants, mean (SD), range** **(*n* = 158)**	**Female, mean (SD), range** **(*n* = 74)**	**Male, mean (SD), range** **(*n* = 84)**	***p*-value**
Full-scale IQ	94.3 (20.4), 49–130	98.9 (18.8), 49–128	90.4 (21.1), 49–130	0.007*
VCI	96.1 (20.9), 49–138	98.9 (19.0), 49–138	93.5 (22.3), 49–133	0.056
VSI	94.1 (17.7), 49–138	95.5 (17.5), 49–124	92.8 (18.0), 49–132	0.298
FRI	93.1 (18.6), 49–133	97.8 (16.8), 49–131	89.0 (19.3), 49–133	0.002*
WMI	97.1 (19.4), 49–137	100.6 (17.7), 49–131	94.0 (20.4), 49–137	0.014*
PSI	91.2 (20.3), 49–126	95.7 (21.2), 49–126	87.2 (18.6), 49–123	4.98 × 10^∧−4*^
VAI	96.6 (19.2), 49–132	98.5 (18.1), 49–132	94.9 (20.0), 49–132	0.197
**Low average scores** **<90, No./Total (%)**
Full scale IQ	55/158 (34.8)	17/74 (23.0)	38/84 (45.2)	0.006*

*VCI, verbal comprehension index; VAI, vocabulary acquisition index; VSI, visual spatial index; FRI, fluid reasoning index; PSI, processing speed index; WMI, working memory index*.

* *Statistically significant between sexes*.

### Diet Quality

HEI-2010 diet quality scores ranged from 19.2 to 87.1, with a mean (SD) of 58.2 (12.4). As previously reported (28), most children had diet scores that fell within the “poor” (43/158 children) or “needs improvement” (106/158 children) diet quality categories. Only 6% of our cohort had HEI-2010 scores that were above 80, indicative of a good quality diet meeting recommended age-specific guidelines. There were no sex differences in HEI-2010 total scores.

### Association Between Exposure and Outcome Variables

Of the 158 children who were tested, 149 children were included in the primary analyses due to missing covariate data (Figure 1). HEI-2010 scores were not significantly associated with IQ scores (Table 3). However, statistically significant variables in our model were birth weight, sex, and maternal education. Every additional gram of birth weight was associated with a gain of 0.02 points in IQ (95% CI, 0.01–0.03). On average, males had IQ scores that were 7.7 points below females (95% CI, 1.5–13.8). The strongest predictor of IQ in our model was maternal education. Children whose mothers had a university degree were found to have an IQ of 9.1 points greater, or a 0.6 standard deviation difference, than children whose mothers were college-educated or had no post-secondary education (95% CI, 1.8–16.2). The interaction between HEI-2010 scores and sex were assessed in the adjusted linear regression model to test whether the association between diet quality and IQ varied by sex. This interaction term, however, was not significant (*p* = 0.83) and therefore removed from the model. Further, the associations between the different components of the HEI-2010 (i.e., total fruits, total vegetables, whole grains, etc.) with IQ were also investigated in adjusted linear regression models but revealed no significant associations (Supplementary Table 3).

**Table 3:** Associations between diet quality, sociodemographic variables and IQ in children born VLBW (*n* = 149).

	**Full-scale IQ**	
**Factor**	**β (S.E.)**	***p*-value**
HEI-2010 score, per point	−0.026 (0.129)	0.839
Birth weight, per g	0.017 (0.006)	0.006^*^
Sex, reference = female	−7.670 (3.105)	0.015^*^
Breastfeeding duration, per day	0.004 (0.009)	0.642
Income below the poverty line, reference = no	−2.595 (4.277)	0.545
Maternal education, reference = no university	9.059 (3.635)	0.014^*^

*Results presented are beta coefficients (standard error) from linear regression models for HEI-2010 scores and IQ adjusted for sex, birth weight, income, maternal education, and breastfeeding duration. HEI-2010, Healthy Eating Index 2010, VLBW, very low birth weight*.

* *Statistically significant variables in regression model*.

Secondary outcome variables (other WPPSI-IV composite scores) were regressed on HEI-2010 scores and revealed non-significant associations (*p* > 0.05). These findings remained unchanged in a sensitivity analysis that excluded children who were unable to complete the assessments and who would have otherwise been assigned a score of 49 (Supplementary Table 4). In sensitivity analyses, in-hospital enteral feed type (e.g., >50% mother's breastmilk, donor milk, or preterm formula) was included in the General linear model (GLM) as a covariate but was not statistically significant.

## Discussion

Our findings demonstrate that diet quality at 5 years of age was not correlated with IQ or any other WPPSI-IV composite measures in children born VLBW. There are no comparable data in the preterm or VLBW populations, but our findings are inconsistent with the few studies who report a positive association between diet quality and cognition in full-term children, even after adjusting for a range of confounding factors (12, 35, 36). Unlike our findings, Khan et al. (36) in a cross-sectional study from the United States assessing diet quality using the HEI-2005 version, found positive associations with executive function in full-term children (*n* = 65, 7–9 years). Similarly, Haapala et al. (35) in a large Finnish cohort of full-term children (*n* = 428, aged 6–8 years), found that poor diet quality assessed using the Baltic Sea Diet Score, was associated in a dose-dependent manner with lower cognitive scores on the Raven's Colored Progressive Matrices; this relation was strongest for the boys. Similar to the HEI-2010, the Baltic Sea diet score assesses diet quality but is specific to Nordic dietary recommendations. Both full-term studies, however, did not directly measure IQ and were performed in older cohorts of children, which may explain why we did not find significant associations between diet quality and cognitive performance in our study (35, 36). Another reason for this discrepancy is the index of diet quality used in these studies, which makes comparison difficult. Further, with the exception of the previously mentioned studies (35, 36), few other studies have used validated measures of diet quality, such as the HEI-2010 in children. Thus, future studies should focus on investigating diet quality using validated tools to better understand its impact on cognition in young children.

In this cohort, the average HEI-2010 score was 58.2 out of 100, with the majority of children (94%) having diets in the “needs improvement” or “poor quality” range. Although diet quality scores were low in our sample of children born VLBW, they are similar to those reported in Khan et al.'s cohort of 7–9-year-old full-term children (mean HEI-2005 score: 66.4) and Florence et al.'s cohort of 10–11-year-old full-term children (mean DQI-I score: 62.4), both of which found significant associations between diet and cognitive performance. However, the lack of variability in our diet scores may be one reason why we did not find significant associations between diet quality and IQ. It is also possible that the association between diet and cognition, may become more apparent with increasing age as IQ scores begin to stabilize over childhood (12, 36). Our results may also indicate that other factors are more critical in supporting neurodevelopment in children born VLBW that were not assessed in this study, such as early growth (9, 37–39). More specifically, it is important to consider the confounding role of SES factors related to neurodevelopment and diet quality in children such as maternal education.

In this cohort, maternal education was the strongest predictor of IQ, with children whose mothers had a university degree, compared to no university degree, having a 9-point gain in IQ. SES indicators such as maternal education have been reported to be predisposing risk factors of preterm birth and have also been linked with adverse cognitive and academic outcomes in both preterm and full-term populations (40, 41). Our findings are similar to those of Voss et al. (41), who reported that 10–13-year-old children born preterm (*n* = 148, mean: birth weight = 798 g, GA = 27.1 weeks) from mothers with low educational background (high school or less) were almost 22 times more likely to have an IQ score ≤ 85 compared to those from more highly-educated mothers (college or higher). The reasons for this association may be due to many factors, including parenting style and quality of cognitive stimulation (41). Given the current and previous findings that maternal education is predictive of cognitive performance in preterm children (42, 43), early interventions focused on supporting families with low maternal education may help attenuate cognitive impairment related to preterm birth.

Despite previous findings that children born preterm are consistently below full-term children in neurocognitive outcomes, encouragingly mean IQ scores were within the average range in our cohort. Males, however, had significantly lower IQ, FRI, WMI and PSI scores compared to females and consistently underperformed in all other cognitive domains. This is in line with previous developmental studies, showing females have a slight IQ advantage in early to mid-childhood due to earlier maturational processes (44, 45). Consistent with this, Lean et al. (46) found males born very preterm were at higher risk of poor intellectual outcomes compared to very preterm-born females at 5 years of age. Thus, future studies and interventions should consider the heightened “triple-risk” of being born male, preterm and to socioeconomically disadvantaged mothers, to mitigate later cognitive risk.

Our study has many strengths, including using the HEI-2010, a measure of diet quality that has been validated in young children (22). In addition, dietary intake was assessed using two 24-h recalls that were adjusted for usual intake using the NCI method. To our knowledge, this is the first study to assess the association between diet quality and cognitive performance in young children born VLBW. Further, our study adjusted for sociodemographic variables such as maternal education and household income, which many other studies fail to take into account. However, despite the many strengths of our study, there are some limitations to consider. Only 50% of the survivors from the original trial participated in this 5-year follow-up. Great efforts were made to contact all 316 families who participated in the initial trial, however, due to various reasons indicated in Figure 1, many families were unable or not interested in participating in this follow-up study. While sample retention is a common challenge faced in prospective cohort studies, we acknowledge that our sample may not be representative of all children born VLBW. However, our sample represented diversity in educational attainment and household income, as well as similar incidence of major morbidity to national data of VLBW infants (47). Another limitation is the lack of a matched control group in our study. Although this study was performed cross-sectionally (i.e., association between diet quality and cognitive performance at 5 years), children were part of a prospective cohort study, from birth, in which they were recruited from birth. Follow-up studies such as ours are costly and time-intensive, making it difficult to include a control group. We did, however, find that average diet quality scores in full-term children were similar to our results (12, 36). In addition, we also acknowledge the possibility of other unmeasured confounding variables, such as the amount and quality of preschool education, that may influence the relations between diet and cognition. We did, however, collect and control for various other sociodemographic factors that could influence neurodevelopment such as sex, household income above/below the poverty line, maternal education, birth weight and breastfeeding duration. Further research is also necessary to assess the impact of specific micronutrients (e.g., iron) and its association with neurodevelopment, which was not assessed in the current study.

Diet quality, assessed using the HEI-2010, was not associated with IQ in 5-year-old children born VLBW. Although this is inconsistent with previous studies reporting a positive association between diet quality and cognition in older cohorts of full-term children, our study is the first to assess this association in 5-year-old children born VLBW. While it is unclear which factors contribute to these discrepant findings, the age range of our cohort, the measure of diet quality used, or the lack of variability in the diet quality scores (most within the “needs improvement” range), all may contribute to these differences. In addition, previous studies did not directly assess the association between diet quality and IQ, which may explain differences with our study findings. Maternal education, however, was independently associated with cognitive performance, and was the strongest predictor of IQ in our model. Maternal education is an important predictor of neurodevelopment in children born VLBW and should be considered in future interventions to target those at risk for cognitive difficulties. Finally, most children in our study had diets that were “in need of improvement”, which has important implications for the health and developmental outcomes in this vulnerable population. Future studies are needed to confirm these findings and assess the impact of diet quality on cognitive performance in young children born VLBW.

## Data Availability Statement

The datasets presented in this article are not readily available because deidentified individual participant data will not be made available in order to protect the privacy and confidentiality of our participants. We do not have consent from participating families to share their anonymized data, nor do we have permission from the research ethics boards of our participating hospitals. Requests to access the datasets should be directed to deborah.oconnor@utoronto.ca.

## Ethics Statement

The Hospital for Sick Children's Research Ethics Board (REB# 1000053053) approved the study protocol. Written informed consent to participate in this study was provided by the participants' legal guardian/next of kin.

## Author Contributions

JS, MM, SU, and DO'C contributed to conception and design of the study. JS, MM, and NB were involved in data collection. JS and MM performed the statistical analysis. JS wrote the first draft of the manuscript. All authors contributed to manuscript revision, read, and approved the submitted version.

## Funding

This study was funded by the Canadian Institutes of Health Research (FHG 129919) and the SickKids Research Institute Restracomp Scholarship to JS.

## Conflict of Interest

The authors declare that the research was conducted in the absence of any commercial or financial relationships that could be construed as a potential conflict of interest.

## Publisher's Note

All claims expressed in this article are solely those of the authors and do not necessarily represent those of their affiliated organizations, or those of the publisher, the editors and the reviewers. Any product that may be evaluated in this article, or claim that may be made by its manufacturer, is not guaranteed or endorsed by the publisher.
