# Supplementary material for: Diet Quality and Cognitive Performance in Children Born Very Low Birth Weight
Source: Front Nutr. 2022 Jul 19;9:874118. doi: 10.3389/fnut.2022.874118 (PMC9343771; doi:10.3389/fnut.2022.874118)
Supplement: Supplementary file 1 [file Data_Sheet_1.docx]

Supplementary Material

**SUPPLEMENTARY TABLE 1.** Components of the Healthy Eating Index 2010 (HEI-2010).

| **HEI-2010 Component** | **Maximum Score** |
| --- | --- |
| **Adequacy (higher score indicates higher consumption)** | |
| Total fruit | 5 |
| Whole fruit | 5 |
| Total vegetables | 5 |
| Greens and beans | 5 |
| Whole grains | 10 |
| Dairy | 10 |
| Total protein foods | 5 |
| Seafood and plant proteins | 5 |
| Fatty acids | 10 |
| **Moderation (higher score indicates lower consumption)** | |
| Refined grains | 10 |
| Sodium | 10 |
| Empty calories | 20 |
| Total score | 100 |

**SUPPLEMENTARY TABLE 2**. The HEI-2010 Components and Standards for Scoring.

| Component  (Maximum points) | Standard for Maximum Score | Standard for Minimum Score |
| --- | --- | --- |
| **Adequacy** | | |
| Total fruit (5) | ≥0.8 cup equiv. per 1000 kcal | No fruit |
| Whole fruit (5) | ≥0.4 cup equiv. per 1000 kcal | No whole fruit |
| Total vegetables (5) | ≥1.1 cup equiv. per 1000 kcal | No vegetables |
| Greens and beans (5) | ≥0.2 cup equiv. per 1000 kcal | No dark green vegetables or greens and beans |
| Whole grains (10) | ≥1.5 oz equiv. per 1000 kcal | No whole grains |
| Dairy (10) | ≥1.3 cup equiv. per 1000 kcal | No dairy |
| Total protein foods (5) | ≥2.5 oz equiv. per 1000 kcal | No protein foods |
| Seafood and plant proteins (5) | ≥2.5 oz equiv. per 1000 kcal | No seafood or plant proteins |
| Fatty acids (10) | (Polyunsaturated +  Monounsaturated)/Saturated ≥2.5 | (Polyunsaturated +  Monounsaturated)/Saturated ≤1.2 |
| **Moderation** | | |
| Refined grains (10) | ≤1.8 oz equiv. per 1000 kcal | ≥4.3 oz equiv. per 1000 kcal |
| Sodium (10) | ≤1.1 gram per 1000 kcal | ≥2.0 grams per 1000 kcal |
| Empty calories (20) | ≤19% of energy | ≥50% total energy |

Adapted from Guenther, et al. (2013). Update of the Healthy Eating Index: HEI-2010. Journal of the Academy of Nutrition and Dietetics 113, 569–580.

**SUPPLEMENTARY TABLE 3.** Associations between the Healthy Eating Index-2010 components and FSIQ (n=149).

|  | **Full-scale IQ (FSIQ)** | |
| --- | --- | --- |
| **Factor** | Adjusted β (S.E.) | *p*-value |
| **Adequacy** | | |
| Total fruit | -2.15 (1.10) | 0.05 |
| Whole fruit | -0.52 (0.95) | 0.58 |
| Total vegetables | 0.38 (1.11) | 0.73 |
| Greens and beans | -0.03 (1.04) | 0.97 |
| Whole grains | 0.83 (0.46) | 0.07 |
| Dairy | -0.05 (0.66) | 0.93 |
| Total protein foods | -0.16 (1.12) | 0.88 |
| Seafood and plant proteins | 0.56 (0.74) | 0.45 |
| Fatty acids | -0.64 (0.50) | 0.20 |
| **Moderation** | | |
| Refined grains | 0.11 (0.49) | 0.82 |
| Sodium | -0.14 (0.51) | 0.79 |
| Empty calories | -0.19 (0.35) | 0.58 |

Data were analyzed using linear regression models that were adjusted for sex, birth weight, income, maternal education, and breastfeeding duration. Results presented are beta coefficients (standard error).

**SUPPLEMENTARY TABLE 4.** Associations between diet quality, sociodemographic variables and IQ, excluding children with imputed scores (n=136).

|  | **Full-scale IQ (FSIQ)** | |
| --- | --- | --- |
| **Factor** | β (S.E.) | *p*-value |
| HEI-2010 score, per point | 0.090 (0.093) | 0.332 |
| Birth weight, per g | 0.012 (0.004) | 0.008 |
| Sex, reference = female | -8.087 (2.208) | <0.001 |
| Breastfeeding duration, per day | 0.004 (0.006) | 0.549 |
| Income below the poverty line, reference = no | -5.036 (3.093) | 0.106 |
| Maternal education, reference = no university | 5.340 (2.651) | 0.046 |

Results presented are beta coefficients (standard error) from linear regression models for HEI-2010 scores and FSIQ adjusted for sex, birth weight, income, maternal education, and breastfeeding duration. HEI-2010: Healthy Eating Index 2010, VLBW: very low birth weight.
